# Global Transcriptome and Weighted Gene Co-Expression Network Analyses of Cold Stress Responses in Chinese Cabbage

**DOI:** 10.3390/genes16070845

**Published:** 2025-07-20

**Authors:** Jizong Zhang, Songtao Liu, Huibin Li, Mengmeng Sun, Baoyue Yan, Peng Zhang, Lifeng Zhang

**Affiliations:** 1 College of Agronomy, Hebei Agricultural University, Baoding 071000, China; zhjz@hebau.edu.cn (J.Z.); lihb@hebau.edu.cn (H.L.); meng77699@163.com (M.S.); 18303075991@163.com (B.Y.); 13659148747@163.com (P.Z.); 2 College of Agriculture and Forestry, Hebei North University, Zhangjiakou 075000, China; 15028293845@163.com

**Keywords:** Chinese cabbage, cold temperatures, RNA-seq, vernalization, WGCNA

## Abstract

Background/Objectives: Chinese cabbage (*Brassica rapa* ssp. *Pekinensis*, AA) growth and development is highly sensitive to cold temperatures. Prolonged low-temperature exposure during early growth stages can induce premature bolting, which reduces market quality and yield. Methods: Here, using comparative leaf RNA-seq transcriptome analysis of plants grown at 6, 9, 12, and 15 °C, we explored key genes and metabolic pathways regulating Chinese cabbage cold response. Results: RNA-seq transcriptome analysis identified a total of 1832 differentially expressed genes (DEGs) in the three comparison groups, with 5452, 1861, and 752 DEGs specifically expressed in the A6_vs_A15, A9_vs_A15, and A12_vs_A15 groups, respectively. KEGG enrichment analysis of DEGs showed that sulfur metabolism, secondary metabolites biosynthesis and photosynthesis pathways were mostly affected by cold stress. K-means clustering revealed distinct expression profiles among the DEGs enriched in cold stress response-associated clusters. Subsequently, DEGs were divided into 18 modules by WGCNA, whereupon co-expression genes that clustered into similar modules exhibited diverse expression and were annotated to various GO terms at different temperatures. Module-trait association analysis revealed M1, M2, M3, and M6 modules as key clusters potentially linked to vernalization-related processes. These modules harbored candidate hub genes encoding transcription factors (including MYB, bZIP, and WRKY), protein kinases, and cold-stress-responsive genes. Additionally, phenotypic analysis showed that 12 °C to 15 °C supported optimal growth, whereas <9 °C temperature inhibited growth. Physiological measurements showed increased antioxidant enzyme activity and proline accumulation at 6 °C. Conclusions: Overall, our study provides a set of candidate cold-stress-responsive genes and co-expression modules that may support cold stress tolerance breeding in Chinese cabbage.

## 1. Introduction

Chinese cabbage (*B. rapa* ssp. *pekinensis*, AA), commonly referred to as Napa cabbage or Peking cabbage, is a leafy vegetable that holds significant culinary and agricultural importance, particularly in East Asia. Belonging to the Brassicaceae family, it is closely related to other staple crops such as broccoli, turnip, and mustard greens [1]. Originally domesticated in East Asia over a thousand years ago, Chinese cabbage has become a global crop due to its adaptability, nutritional value, and versatility in cooking. The vegetable is characterized by its dense, elongated, pale green leaves that form a tight, barrel-shaped head, and it is prized for its mild, slightly sweet flavor and crunchy texture [2].

Agronomically, Chinese cabbage is highly sensitive to temperature, which can significantly impact its growth, physiology, and development. However, exposure to prolonged cold, especially temperatures below 10 °C (50 °F), can induce vernalization, a process that prepares the plant for flowering and seed production [3]. Prolonged cold triggers the cabbage to transition from vegetative growth to reproductive development, potentially leading to premature flowering (bolting). This premature bolting reduces the market value of the cabbage, by compromising both its appearance and taste [4,5]. In response, growers have developed methods to manage the effects of low temperatures, such as selecting cold-resistant varieties that are less sensitive to vernalization and implementing protective measures like seedling transplantation to minimize exposure [6]. In response to low temperatures, Chinese cabbage also undergoes biochemical changes, increasing the production of certain protective compounds like antioxidants and glucosinolates, which can contribute to enhanced nutritional properties [7,8]. Understanding and managing the effects of cold is thus crucial for optimizing both yield and quality in regions where cold temperatures are prevalent during the growing season.

The northwestern region of Hebei Province lies along the southern edge of the Inner Mongolia Plateau, with high altitudes and a cool climate, offering unique advantages for producing summer-grown plateau vegetables. Chinese cabbage has been widely cultivated in this region since the mid-1990s, following the successful development of open-field direct seeding techniques that prevent premature bolting. Subsequently, different types of Chinese cabbage were identified based on their vernalization temperatures, including cold-insensitive, semi-insensitive, and sensitive varieties [9]. This progress, along with seedling transplantation techniques to prevent bolting, has significantly advanced the crop’s market availability date [10]. Over nearly 30 years of development, the area dedicated to cool-loving vegetables, primarily Chinese cabbage, has expanded to over 40,000 hectares, establishing this region as China’s fifth-largest vegetable production base [8,11]. Although non-bolting and low-temperature vernalization techniques for Chinese cabbage have been revolutionized in this region. The physiological and biochemical mechanisms underpinning the tolerance of main local non-bolting spring varieties to vernalization remain unclear.

RNA sequencing (RNA-Seq) is a powerful and widely used technology for examining and quantifying RNA molecules within a biological sample, providing a comprehensive snapshot of the transcriptome, and allowing researchers to analyze gene expression levels, discover novel transcripts, and detect alternative splicing events, all at unprecedented scale and resolution [12,13]. Due to its advantages (low cost, high-throughput, and high sensitivity) over traditional sequencing methods, RNA-seq has been central in several transcriptomic studies focused on revealing important abiotic stress response mechanisms in plants [14,15,16,17]. Meanwhile, weighted gene co-expression network analysis (WGCNA) serves to identify subsets of genes that are highly correlated with each other within the network. This method has become an essential tool for discovering gene co-expression modules and examining the functional relationships between these gene networks and associated traits [18,19]. WGCNA offers a system-level view of gene expression data, whereby genes are grouped into co-expression modules based on their expression profiles across samples. These modules are then analyzed for biological relevance, helping to reveal core regulatory mechanisms and identify potential biomarkers [16].

WGCNA has been used to reveal hub genes and key pathways underpinning crop response and tolerance to abiotic and biotic stresses including vernalization in Chinese cabbage [20], low temperature in maize seedlings [19], cold stress in cotton [21], and smut (*Sporisorium scitamineum*) resistance in sugarcane [22]. For instance, shoot system morphogenesis and polysaccharide and sugar metabolism pathways were found to trigger early and quicker bolting and flowering, whereas hub genes *MTHFR2*, *CPRD49*, *AAP8*, *BXLs*, *endoglucanase 10*, *WRKY*s, and *GATL*s were highly connected in regulating vernalization in *B. rapa* [20]. Earlier, transcripts linked to plant hormone signal transduction, starch and sucrose metabolism, photoperiod and circadian clock, as well as vernalization pathways had been observed to change remarkably in *B. rapa* at different vernalization periods, with associated genes such as *TPS*, *UGP*, *CDF*, *VIN1,* and seven hormone pathway genes exhibiting different expression patterns [23]. Vernalization up-regulated the transcription of floral transition and glucosinolate (GSL) pathway genes and modulated glucosinolate biosynthesis in *B. rapa*, suggesting that GSL biosynthesis pathway is important for cold stress response in Chinese cabbage [24]. Meanwhile, *FLOWERING LOCUS C* (*FLC*) gene is a key component of the vernalization pathway which controls flowering time. Several upstream genes govern bolting and flowering time by modulating *FLC* expression [25]. *FLC* encodes a MADS-box transcription factor, which is a flowering inhibitor, and the *FLC* allele variation mostly determines the difference between early and late flowering [20,25]. In addition, competitive endogenous RNA (ceRNA) networks [26,27] and epigenetic regulation mechanisms, such as DNA methylation of *BrCKA2* (catalytic α-subunit of casein kinase II) and *BrCKB4* (regulatory β-subunit of CK2) [28], regulate vernalization and flowering in Chinese cabbage [29]. Therefore, identifying the key interacting gene partners and vernalization pathway-associated networks is crucial for establishing the key mechanisms underpinning bolting and flowering in Chinese cabbage.

To elucidate the molecular mechanisms underlying cold stress responses in Chinese cabbage, we conducted an in-depth comparative transcriptome analysis using leaf tissues from the local spring cultivar Linglong Yellow No. 2 (LY2), cultivated for 30 days under four precisely regulated temperature conditions: 6 °C, 9 °C, 12 °C, and 15 °C. Complementary physiological assessments were also performed to integrate transcriptomic and phenotypic data, offering a systems biology perspective on cold adaptation. Furthermore, WGCNA enabled the identification of gene modules with co-regulated expression patterns closely associated with cold-responsive metabolic pathways. These results contribute valuable insights into the genetic regulation of cold tolerance in *B. rapa*, and lay the groundwork for future functional genomics and gene cloning studies targeting stress-resilient traits.

## 2. Materials and Methods

### 2.1. Plant Materials and Cold (Low-Temperature) Treatment Conditions

**Figure 1:**
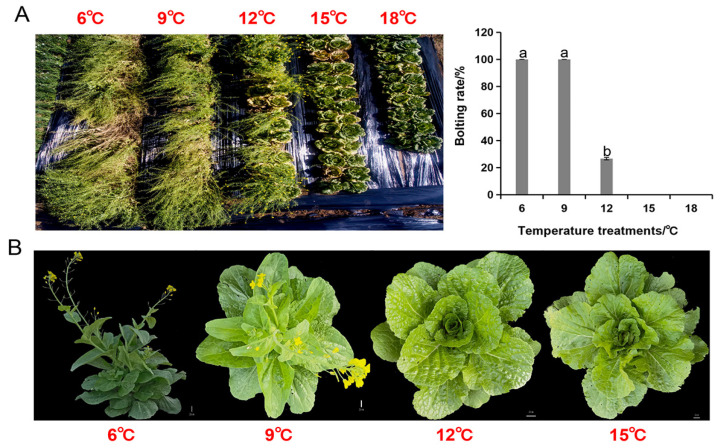
Phenotypic response of Chinese cabbage variety LY2 to cold stress. (**A**) Bolting status of Chinese cabbage under different temperature treatments in the field. (**B**) Performance of Chinese cabbage under varying temperature treatments. Different lowercase letters indicate significant differences between groups at *p* < 0.05.

In this experiment, we used a local Chinese cabbage variety Linglong Yellow No. 2 (LY2). To ensure genetic consistency across all treatments, seeds were provided by our lab (Zhangbei Experimental Station, Hebei Agricultural University, China). To determine the treatment temperatures, we first conducted a field experiment whereby bolting situation was investigated under 6, 9, 12, 15, and 18 °C growth conditions (Figure 1A). Undamaged seeds were surface sterilized with 1% sodium hypochlorite for ten minutes, followed by sterile water wash three times. The seeds were sown in uniform PVC pots (10 cm × 9.5 cm), at three seeds per pot. The pots were filled with homogenous potting soil, and each treatment comprised sixty pots. The seedlings were nurtured in a controlled growth chamber for 15 days under the following conditions: 14 h light/10 h dark photoperiod, 25 °C temperature, and 60 ± 5% relative humidity. Thinning was carried out twice—once after cotyledon expansion and again before temperature treatment. After thinning, one healthy seedling with uniformly developed second true leaves was retained per pot. The seedlings were then transferred to growth chambers under constant temperatures of 6 °C, 9 °C, 12 °C, 15 °C, and 18 °C, respectively, and nurtured for an additional 30 days under controlled conditions. Upon the completion of the temperature treatments, uniformly growing plants were selected from each group. To reduce tissue-specific transcriptomic variation, we collected one fully expanded, uniformly developed functional leaf from each plant. Entire leaf blades (excluding petioles) were harvested, and every five leaves were pooled as one biological replicate for RNA extraction. This approach ensured sampling consistency across all treatments. Samples were immediately liquid-nitrogen-frozen and stored at –80 °C for subsequent transcriptome sequencing and quantitative real-time PCR (qRT-PCR) analyses. Three biological replicates were performed for each temperature treatment.

Subsequently, 40 uniformly growing seedlings from each treatment group were transferred into an experimental field located in northwestern Hebei Province. All transplants were grown under the same field conditions (including temperature which was at an average of ~18 °C), and the local agronomic practices for high-yield Chinese cabbage production were adopted. After 60 days of field cultivation, when the leafy heads were fully developed and harvestable, the bolting rate was investigated. Plants were considered bolted if the visible flower stalk or the elongated shoot apical meristem exceeded 0.5 cm in length. In case of no visible bolting, the leafy heads were longitudinally dissected with a knife to examine the shoot apical meristem. Plants with elongated meristems were classified as bolted, and those with unelongated meristems were classified as non-bolted.

The analysis of the field-grown treatment groups showed that bolting rate was zero for the plants that had been pretreated with >15 °C temperature. Therefore, four temperature conditions (6 °C, 9 °C, 12 °C, and 15 °C, with 15 °C taken as non-vernalization or control temperature treatment) were selected for RNA-Seq analysis to identify genes associated with vernalization tolerance in Chinese cabbage (Figure 1B).

### 2.2. Phenotypic and Physiological Characterizations

Phenotypic and physiological characterizations of LY2 cultivar under different temperatures were carried out. Plant height was measured, from the base of the stem (soil level) to the highest point of the plant (the apex of the innermost leaf), using a graduated ruler. Basal stem diameter (2–3 cm above the soil surface) was measured using a vernier caliper. Leaf length (from leaf base to tip) and leaf width (perpendicular to the midrib) were measured using a ruler, and the leaf area (cm^2^) calculated by the following formula: length (cm) × width (cm) × correction factor.

Peroxidase (POD) and catalase (CAT) enzyme activities were determined using the guaiacol substrate and decomposition of hydrogen peroxide (H_2_O_2_) methods, respectively. Proline (Pro) content was determined using the acid ninhydrin assay. Leaf chlorophyll content was measured using a SPAD-502 meter (Konica Minolta, Tokyo, Japan), whereby ten mature leaves per plant were sampled, and their SPAD values averaged to represent chlorophyll content. Electrical conductivity was determined through immersion of three leaf disks (10 mm diameter) in 15 mL deionized water. After shaking incubation at 25 °C for 2 h, initial conductivity (C_1_) was measured using a DDSJ-308A conductivity meter (INESA Scientific Instrument, Shanghai, China). The samples were then boiled at 100 °C for 30 min and final conductivity (C_2_) recorded. Relative electrolyte leakage (%) was calculated using the formula (C_1_/C_2_) × 100. Meanwhile, the leaf soluble sugar content was measured through the anthrone-sulfuric acid method [30].

### 2.3. Total RNA Extraction, cDNA Library Construction, and Transcriptome Analysis

Total RNA was extracted from leaf samples using the TRIzol reagent (Invitrogen, Carlsbad, CA, USA), following the manufacturer’s protocol. RNA concentration and purity were assessed using a NanoDrop 1000 spectrophotometer (NanoDrop Technologies Inc., Wilmington, DE, USA), while integrity was evaluated via 1% agarose gel electrophoresis. High-quality RNA samples were then submitted to Genedenovo Biotechnology Co., Ltd. (Guangzhou, China) for cDNA library preparation and transcriptome sequencing. Libraries were constructed using the NEBNext^®^ Ultra™ RNA Library Prep Kit for Illumina^®^ (NEB, Ipswich, MA, USA), and paired-end sequencing was performed on the Illumina HiSeq 6000 platform.

### 2.4. Sequencing Reads Processing, Genome Mapping, and Gene Expression Quantification

The raw sequencing reads in FASTQ format, obtained from the Illumina HiSeq 6000 platform, were initially filtered using custom Perl scripts to remove low-quality sequences. Clean reads were subsequently aligned to the *B. rapa* reference genome (version 3.1, http://www.brassicadb.cn/ accessed on 15 May 2025) using TopHat v2.0.12, a splice-aware aligner capable of accommodating intronic regions with gaps of up to 50 kb. Only reads possessing a perfect match or one mismatch were further analyzed and annotated based on the reference genome. Additionally, the read numbers that mapped to each gene were counted by HTSeq v 0.6.1 software. Gene expression levels were quantified using the FPKM (fragments per kilobase of transcript per million mapped reads) method, which normalizes for both transcript length and sequencing depth. Functional annotation of the assembled transcripts was performed through sequence alignment against several major public databases, including the NCBI non-redundant protein database (Nr), Swiss-Prot, Cluster of Orthologous Groups (COG), Kyoto Encyclopedia of Genes and Genomes (KEGG), and Gene Ontology (GO), using the BLAST algorithm (version 2.12.0).

### 2.5. Differentially Expressed Genes (DEGs) Detection, Functional Enrichment Analysis, and Identification of Key Cold-Responsive DEGs

Analysis of gene differential expression was conducted using the DESeq R package (1.10.1) [31], whereby the ratios of FPKM values between the control and treatments were evaluated, and the resulting *p*-values were adjusted for multiple by applying the Benjamini and Hochberg method [32]. In this study, genes with fold change (FC) ≥ 2 and Q-value < 0.05 were assigned as differentially expressed.

For key cold or vernalization-responsive DEGs identification, we first performed gene differential expression analysis of transcripts derived from leaf samples from low (cold) temperature (6 °C, 9 °C, and 12 °C) versus control/non cold (15 °C) temperature, creating three important comparison groups (A6_vs_A15, A9_vs_A15, and A12_vs_A15). Then, the total DEGs derived from these three comparison groups and fitting within the selection criteria specified above (FC ≥ 2 and Q-value < 0.05) were further screened by way of Venn diagram analysis to yield key candidate cold/vernalization-responsive DEGs The DEGs from these important regions were further filtered by analyzing their gene ontology functional annotations, associated KEGG metabolic pathways, and their previously reported roles [33].

To further explore the biological significance of DEGs, KEGG pathway enrichment analysis was performed to identify significantly enriched functional categories and metabolic pathways. In addition, DEGs exhibiting similar expression trends were grouped via K-means clustering, based on log-transformed fold-change values, using the R statistical environment. The optimal number of clusters and associated Q-value threshold (<0.5) were determined using the default parameters. Subsequently, co-expression network construction was carried out using the WGCNA package (version 1.47) in R. Functional enrichment analyses, including GO and KEGG annotations, were applied to genes within each co-expression module to elucidate their potential biological roles.

### 2.6. Quantitative Real Time-PCR (qRT-PCR) Analysis

To validate the RNA-seq data, qRT-PCR was conducted on 15 genes randomly selected for expression analysis. Gene-specific primers were designed using Primer Premier 5 software (Premier Biosoft International, Palo Alto, CA, USA). Total RNA of high integrity was isolated from Chinese cabbage samples and subsequently reverse-transcribed into cDNA using the HiFiscript cDNA Synthesis Kit (CWBIO, Beijing, China) following the manufacturer’s instructions. qRT-PCR program was run in a Bio-Rad iQ5 Thermo Cycler (Bio-Rad, Hercules, CA, USA) using 2 × Fast Super Evagreen qPCR mastermix (US Everbright Inc., Daly City, CA, USA), where fluorescence intensity directly corresponded to the DNA quantity present. A stable reference gene *ACTIN* was used as an internal control for data normalization. The relative mRNA abundance for each gene was determined by the 2^−ΔΔCT^ method [34].

### 2.7. Statistical Analysis of Physiological Data

Statistical analyses were conducted using SPSS software (version 22.0; IBM Corp., Armonk, NY, USA), and results are expressed as mean ± standard error (SE). For physiological measurements, two-way analysis of variance (ANOVA), followed by least significant difference (LSD) post hoc tests, was applied to assess differences among treatments and genotypes. In contrast, the qRT-PCR expression data were analyzed using one-way ANOVA, with Duncan’s multiple range test employed for pairwise comparisons. A significance threshold of *p* < 0.05 was adopted throughout.

## 3. Results

### 3.1. Summary and Quality Assessment of RNA-Seq Results

**Figure 2:**
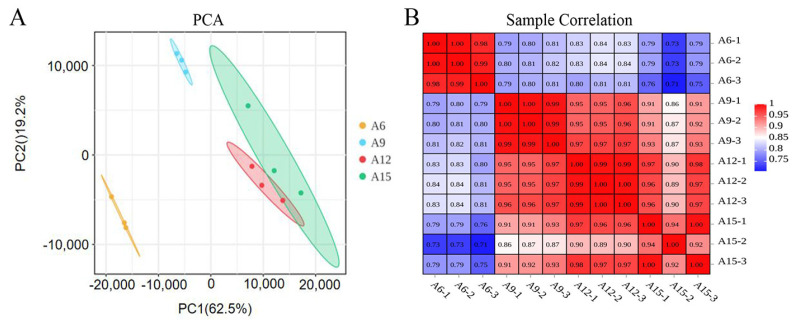
Intersample analysis of 12 leaf tissue samples used for RNA-seq. (**A**) PCA, where there are relative coordinate points on the principal component after the samples are analyzed by dimension reduction. The distance of each sample point from the other represents the relatedness or closeness of that sample to the other. (**B**) Analysis of correlation coefficients between samples.

To clarify the temperature-induced effect and molecular genetic mechanism underlying vernalization in Chinese cabbage, RNA-seq analysis was performed on 12 leaf samples of 30-day-old Chinese cabbage plants (cultivar LY2) grown under four temperature treatment conditions (6, 9, 12, and 15 °C), respectively. The outcomes of the principal component analysis (PCA) indicated a low level of consistency across the three replications of sample A15, likely attributed to a technical issue (Figure 2A). Consequently, the sequencing data were evaluated by utilizing sample A15-2. Pearson correlation coefficients between samples were also calculated and displayed in the form of a heatmap. The results showed each R^2^ (between the two samples) to be higher than 90% (Figure 2B). Overall, these results showed that our experiment is reproducible and reliable, meeting the demands for further analyses.

Following quality assessment, eleven RNA-seq libraries were retained for transcriptomic analysis after excluding one low-quality sample (A15-2). Post-filtering, a total of approximately 49.12 million clean reads were generated from the selected samples. Of these, 90.24% to 91.11% were uniquely mapped to the *B. rapa* reference genome (brassicadb.cn v3.1), while 2.37% to 3.05% were aligned to multiple genomic loci. The base quality metrics were high, with Q30 scores exceeding 92.70% and GC content ranging above 47.48% (Table 1). The paired-end reads had an average length of approximately 150 bp (Figure S1), indicating the generation of high-quality sequencing data suitable for downstream analysis.

### 3.2. Gene Differential Expression Analysis

**Figure 3:**
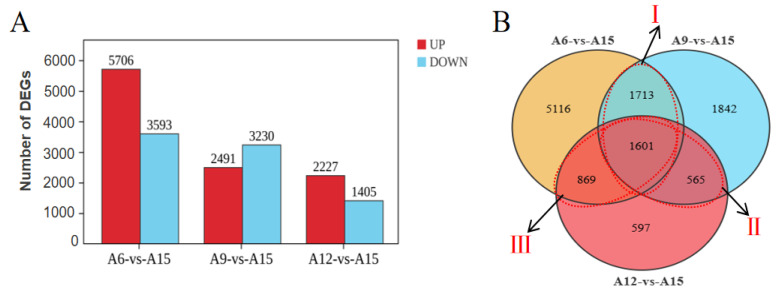
Analysis of differentially expressed genes (DEGs) for vernalization tolerance in Chinese cabbage. (**A**) Number of DEGs identified in each comparison group, where Up represents up-regulated genes, and Down represents down-regulated genes. (**B**) Venn plot analysis of DEGs identified in three groups.

Gene differential expression analysis was performed on leaf samples collected from plants grown at 6 °C, 9 °C, and 12 °C versus 15 °C. Most numbers of DEGs were obtained in A6_vs_A15 group, including 5706 up-regulated and 3593 down-regulated. Meanwhile, a total of 5721 DEGs were identified in group A9_vs_A15, with 2491 up-regulated and 3230 down-regulated. Similarly, we found 3632 DEGs (2227 up-regulated and 1405 down-regulated) in group A12_vs_A15 (Figure 3A). The number of DEGs showing overlaps and specific in different temperatures is visualized in Figure 3B. A total of 1601 DEGs were identified in the three comparison groups, and 5116, 1842, and 597 DEGs were specifically expressed in A6_vs_A15, A9_vs_A15, and A12_vs_A15 groups, respectively. Area I represents 3314 DEGs shared between A6_vs_A15 and A9_vs_A15, area II represent 2170 DEGs shared between A6_vs_A15 and A12_vs_A15, whist area III represent 2470 DEGs shared between A9_vs_A15 and A12_vs_A15.

### 3.3. KEGG Metabolic Pathways Enrichment Analysis of the DEGs

**Figure 4:**
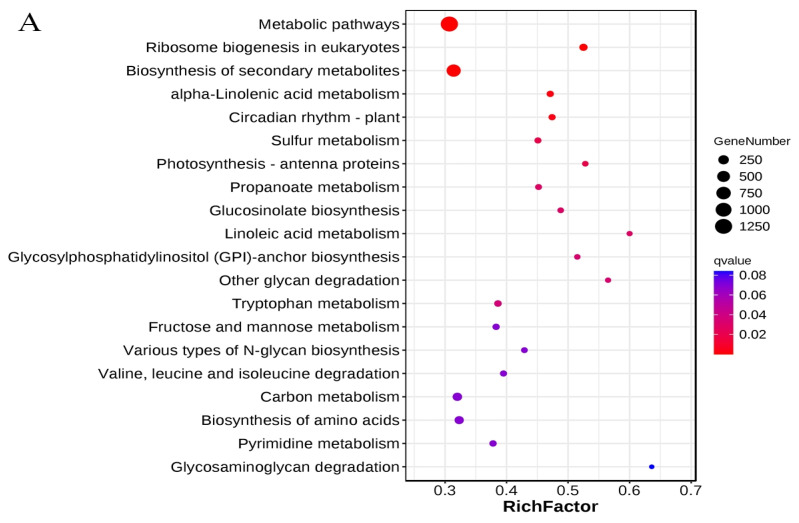

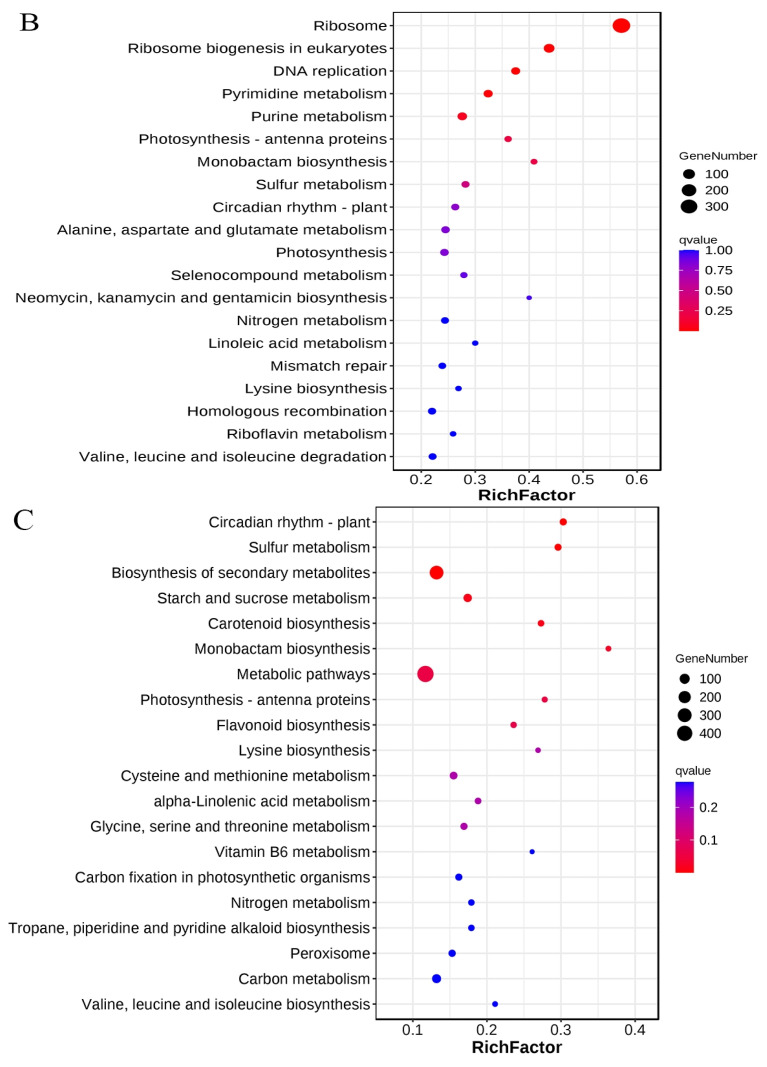
KEGG pathway enrichment analysis of the DEGs related to vernalization in Chinese cabbage. The sub-figures show the most significantly enriched pathways in (**A**) A6_vs_A15; (**B**) A9_vs_A15, and (**C**) A12_vs_A15 comparisons, according to hypergeometric test. The color gradient represents the size of *q* value; the Rich Factor shows the ratio of the number of the DEGs to the total gene number in certain pathways.

KEGG enrichment analysis of DEGs identified in three comparative groups revealed pathways related to metabolic processes to be significantly enriched across all three groups. Sulfur metabolism and photosynthesis—specifically antenna proteins—were highly enriched in three comparison groups (Figure 4). Metabolic pathways and biosynthesis of secondary metabolites were highly enriched in A6_vs_A15 and A12_vs_A15 groups (Figure 4A,C). On the other hand, ribosome and ribosome biogenesis were significantly enriched in the A9_vs_A15 comparison group (Figure 4B). At the same time, the pathways related to stress response, including “starch and sucrose metabolism”, “photosynthesis”, and “nitrogen metabolism”, were significantly enriched in the comparison groups (Figure 4A–C), revealing their possible role in cold stress response in Chinese cabbage.

### 3.4. K-Means Clustering Analysis of DEGs

**Figure 5:**
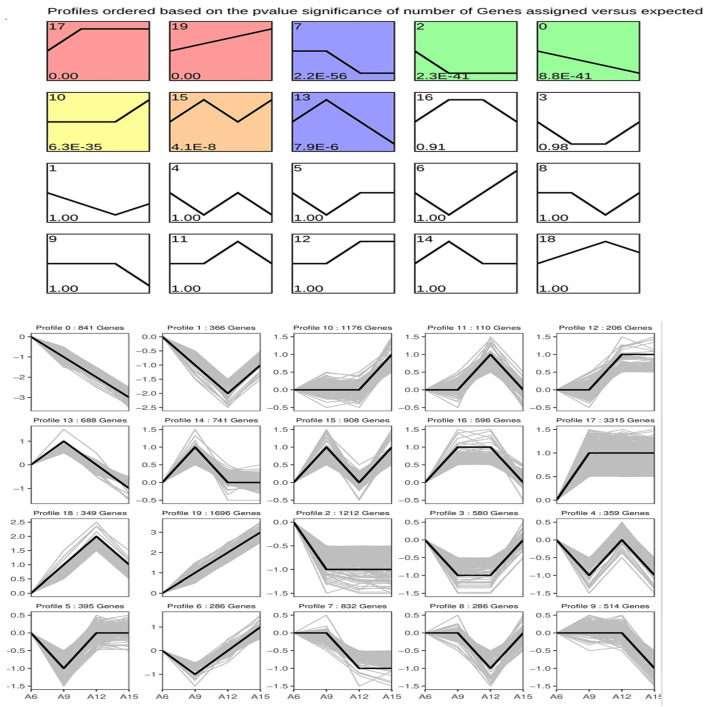
K-means clustering analysis of DEGs during cold treatment. Colored boxes highlight significantly enriched expression profiles based on *p*-value significance of the number of genes assigned versus expected.

K-means clustering analysis was conducted to identify gene clusters exhibiting distinct expression profiles in Chinese cabbage. Subsequently, GO and KEGG enrichment analyses were conducted to explore the biological functions of these special gene clusters. The DEGs identified from the four temperature treatments were grouped into 20 clusters with different expression patterns. The clusters consisted of between 110 and 3315 co-expressed DEGs. Among these, eight gene clusters showed statistically significant expression differences (*p*-value < 0.05) (Figure 5).

Cluster 17 included 3315 DEGs, exhibiting low expression only at 6 °C, and consistently high expression at 9, 12, and 15 °C. These DEGs were significantly enriched in small molecule metabolic processes, lipid biosynthetic processes, and organic acid metabolic processes (Figure 5 and Figure S1A). The 1696 DEGs in cluster 19 showed a gradually increased in expression with rising temperature and were significantly enriched in photosynthesis, carbohydrate derivative metabolic processes, and sulfur compound biosynthetic processes (Figure S1B). In contrast, the 832 DEGs in cluster 7 were highly expressed at 6 and 9 °C, but showed low expression at 12 and 15 °C. These DEGs were enriched in RNA modification, ribosome biogenesis, and ribonucleoprotein complex biogenesis (Figure S1C). The 1212 DEGs in cluster 2 showed high expression at 6 °C and low expression at 9, 12, and 15 °C, following a consistent pattern. These genes were significantly enriched in ion transport, response to abiotic stress, and transmembrane transport (Figure S1D). The 841 DEGs in cluster 0 showed a gradual decline in expression with increasing temperature and were significantly enriched in small molecule metabolism, carboxylic acid biosynthetic process, and small molecule biosynthetic process (Figure S1E). The number of DEGs, expression profiles, and enriched GO terms or KEGG pathways for other gene clusters are shown in Figure S1F–H. Overall, the clustering of DEGs with similar expression patterns and associated GO terms helps in revealing key hub genes and metabolic pathways related to cold stress in Chinese cabbage.

Meanwhile, KEGG enrichment analysis was performed on clusters that were significantly enriched (*q*-value < 0.05), revealing that clusters 0, 2, 17, and 19 were significantly associated with metabolism-related pathways (Figure S2). Specifically, cluster 0 was significantly enriched in cysteine and methionine, biosynthesis of secondary metabolites, and sulfur metabolism. Cluster 2 was significantly enriched in the biosynthesis of secondary metabolites and glycerophospholipid metabolism. Cluster 19 was significantly enriched in metabolic pathways, photosynthesis, and glucosinolate biosynthesis. The metabolic pathway enrichment of other gene clusters is shown in Figure S2. Taken together, metabolic pathways enrichment analysis of these gene clusters helps in identifying key pathways related to cold stress or vernalization response in Chinese cabbage, whose components can be further dissected to reveal the mechanisms underpinning cold stress response in Chinese cabbage.

### 3.5. WGCNA of Differentially Expressed Genes

**Figure 6:**
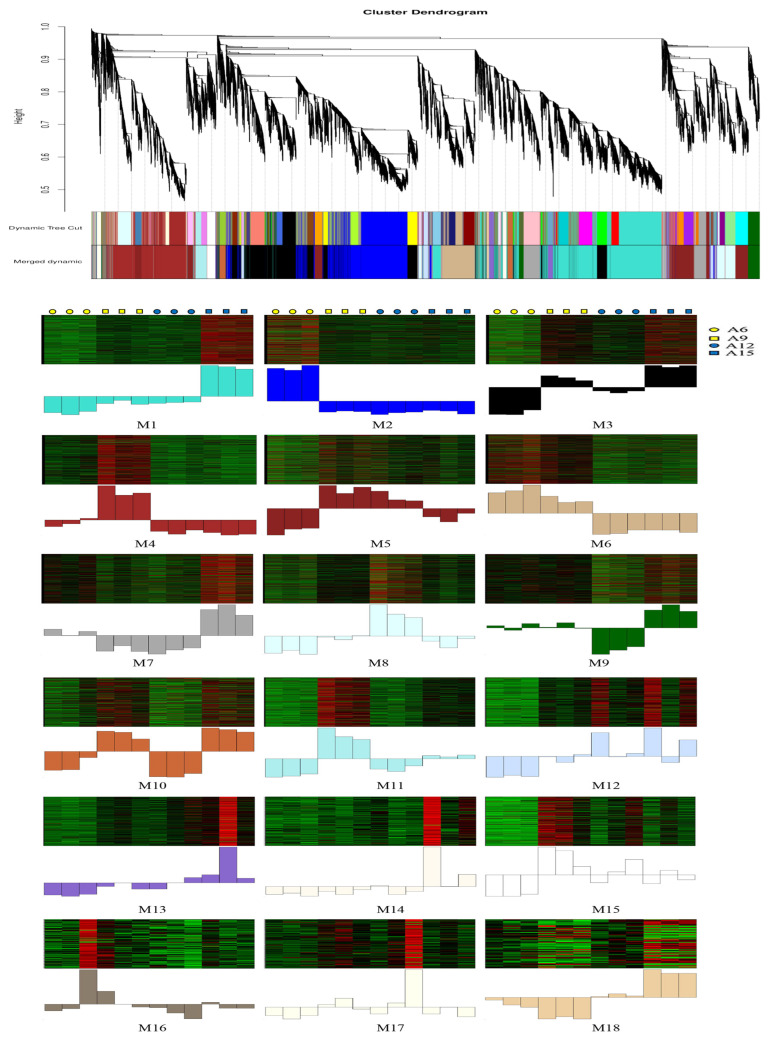
Co-expression network analysis identifying gene modules underlying Chinese cabbage cold stress response at four different growth temperatures.

WGCNA was further applied to analyze the co-expression relationship between genes based on their expression patterns. After filtering, a total of 19,442 DEGs were divided into 18 modules (M1-M18), consisting of between 60 and 3764 co-expressed DEGs (Figure 6). A large number of transcription factor (TF) families were identified to be differentially expressed, with M1 containing the most TFs at 227, followed by M2 with 209 TFs (Table 2). TFs regulation plays a crucial role in plant growth and stress response. In this study, TFs such as HD-Zip, bHLH, bZIP, NAC, MYB-related, MYB, WRKY were identified to have different expression patterns at different growth temperatures (Table S1), and were speculated to play significant roles in cold stress response in Chinese cabbage.

GO enrichment analysis of DEGs from each distinct expression module revealed a range of key biological processes involving co-expression genes. Module 1 (M1), the largest gene cluster with 3764 DEGs, exhibited high expression exclusively at 15 °Cand was significantly enriched in vesicle-mediated transport, endomembrane system, and protein binding (Table 3). The 3350 DEGs of M2 showed high expression at 9 and 15 °C, and low expression at 6 and 12 °C, and significantly enriched in small molecule metabolic processes, response to abiotic stimuli, and ion transport. The 2824 DEGs of M3 were uniquely highly expressed at 6 °C and significantly enriched in localization, transport, and endoplasmic reticulum. The 1133 DEGs of M6 showed elevated expression at 6 and 9 °C, and reduced expression at 12 and 15 °C, and significantly enriched in ribosome biogenesis, ribonucleoprotein complex biogenesis, and rRNA metabolic processes. The 1047 DEGs of M7 were lowly expressed at 9 and 12 °C, whist highly expressed at 6 and 15 °C, was enriched in response to temperature stimuli, response to abiotic stimuli, photosynthesis, and dark reactions. The enriched GO terms for the DEGs of other co-expression modules are shown in Table 3.

### 3.6. Identification of Hub Genes Associated with Vernalization

Many metabolic processes are intricate, resulting from both the actions of single genes and the interactions among combinations of genes. A sizeable portion of the genes in each network module exhibited extremely high connectivity with other genes belonging to other modules, and were designated as hub genes. Owing to their central position within the network clusters, the hub genes were considered to be vital components of the networks. Therefore, we conducted a Network Analyzer-based analysis and found that 5% of the genes in the modules had different expression trends at 15 °C, and could thus be identified as hub genes and chosen for further study (Table S2).

According to the gene classification results, 39 TFs from the 650 hub genes were identified, and they belonged to distinct families, such as MYB (*BraA08g032880.3.1C*), MYB-related (*BraA06g006620.3.1C*), Trihelix (*BraA05g037430.3.1C*, *BraA06g000940.3.1C*), bHLH (*BraA01g034280.3.1C*, *BraA01g020280.3.1C*), bZIP (*BraA03g003370.3.1C*), and AP2 (*BraA03g022150.3.1C*), among others. The core genes with high connection were also identified, including peroxygenase (*BraA04g024980.3.1C*), probable protein phosphatase 2C (*BraA02g042430.3.1C*), protein kinase (*BraA06g038980.3.1C*, *BraA01g042790.3.1C*, *BraA08g024580.3.1C*, *BraA01g024410.3.1C*), photosystem-related (*BraA03g044100.3.1C*, *BraA03g033980.3.1C*, *BraA05g026750.3.1C*, *BraA10g005500.3.1C*, *BraA09g016760.3.1C)*, and CBF (*BraA06g039260.3.1C*, *BraA02g029450.3.1C*) among others (Table S2).

### 3.7. Association of Modules with Phenotypic and Physiological Traits

**Figure 7:**
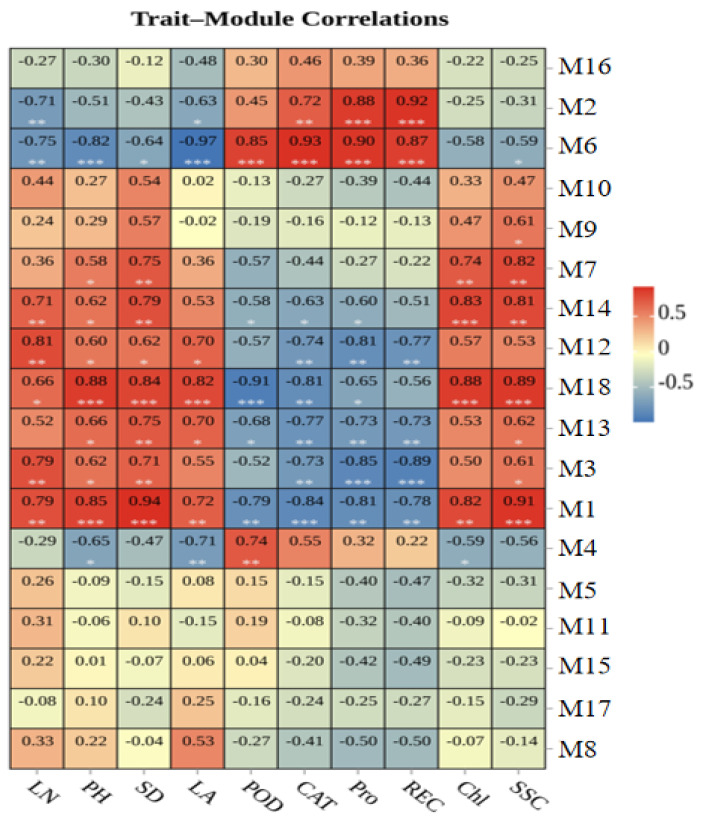
The module–trait relationships of genes involved in phenotypic and physiological traits.

The identification of genes and modules which are associated with phenotypic and physiological characteristics greatly aids our understanding of the underlying mechanisms of certain traits. In our analysis, the leaf number (LN), plant height (PH), stem diameter (SD), leaf area (LA), POD activity, CAT activity, Pro content, relative electrical conductivity (REC), chlorophyll content (Chl), and soluble sugar accumulation (SSC) were selected in the module–trait relationship analysis. As shown in Figure 7, module M6 showed significant negative correlations with phenotypic characteristic LN (r = −0.75, *p* = 0.005), PH (r = −0.82, *p* = 0.001), SD (r = −0.64, *p* = 0.03), and LA (r = −0.97, *p* = 8 × 10^−8^). Meanwhile, M1 showed significant positive correlations with PH (r = 0.85, *p* = 1 × 10^−4^) and SD (r = 0.94, *p* = 7 × 10^−4^). Module M12 and M18 also showed significant positive correlations with LN (r = 0.81, *p* = 0.001) and LA (r = 0.82, *p* = 0.001), respectively.

Module M6 showed significant positive correlations with physiological characteristic POD (r = 0.85, *p* = 4 × 10^−4^), CAT (r = 0.93, *p* = 1 × 10^−5^), Pro (r = 0.90, *p* = 6 × 10^−5^), and REC (r = 0.87, *p* = 3 × 10^−4^). At the same time, M2 and REC (r = 0.92, *p* = 3 × 10^−5^), M18 and Chl (r = 0.88, *p* = 1 × 10^−4^), and M1 and SSC (r = 0.91, *p* = 4 × 10^−5^) showed positive correlations. Module M3 showed significant negative correlations with Pro (r = −0.85, *p* = 4 × 10^−4^) and REC (r = −0.89, *p* = 1 × 10^−4^). Modules M18 and POD (r = −0.91, *p* = 3 × 10^−5^) and M1 and CAT (r = −0.84, *p* = 6 × 10^−4^) also showed significant negative correlations.

### 3.8. Phenotypic and Physiological Responses of Chinese Cabbage to Cold Stress at Different Temperature Treatments

**Figure 8:**
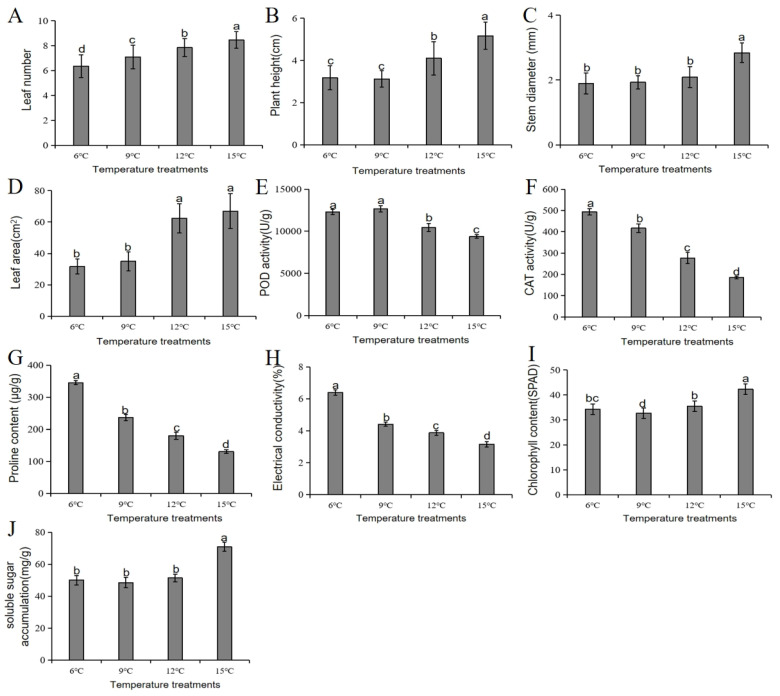
Phenotypic and physiological responses of Chinese cabbage cultivar LY2 to different cold (low temperature) stress conditions. (**A**) Leaf number; (**B**) plant height; (**C**) stem diameter; (**D**) leaf area. (**E**) POD activity; (**F**) CAT activity; (**G**) proline content; (**H**) electrical conductivity; (**I**) chlorophyll content; and (**J**) soluble sugar accumulation. Data are presented as mean ± SE of mean (*n* = 10). Different letters above bar graphs show significant difference (*p* < 0.05) between or among different temperature treatments.

The growth performance of Chinese cabbage was significantly influenced by low-temperature treatment. Plants grown at 15 °C had the highest number of leaves, plant height, stem diameter, and leaf area (Figure 8A–D), indicating optimal vegetative growth at this temperature, and suggesting that moderate-to-warm temperature enhances photosynthetic capacity and biomass accumulation in Chinese cabbage. In contrast, lower temperatures (6 °C and 9 °C) resulted in stunted growth, with significantly reduced leaf numbers and plant heights, and delayed leaf expansion (Figure 8A–D). These findings suggest 12–15 °C as the optimal temperature range for spring Chinese cabbage growth during early growth stages, whilst <9 °C temperatures severely limit growth.

Physiologically, Chinese cabbage exhibited significant temperature-dependent variations across key stress- and growth-related parameters. Lower temperatures (6 °C) triggered pronounced cold stress response, characterized by elevated antioxidant enzyme activities and increased proline accumulation, indicative of enhanced oxidative stress mitigation and osmotic adjustment (Figure 8E–G). As temperature rose to 15 °C, antioxidant activities progressively declined, paralleled by reduced relative electrical conductivity and proline content, suggesting alleviated cell membrane damage and cellular stress (Figure 8H). Notably, 15 °C optimized photosynthetic performance, yielding the highest chlorophyll content and soluble sugar accumulation (Figure 8I–J), reflecting improved carbon assimilation and energy storage under “warmer” temperatures. Intermediate temperatures (9–12 °C) displayed transitional patterns, with partial stress adaptation and suboptimal growth metrics. Collectively, these results reveal 15 °C as the optimal temperature for spring Chinese cabbage growth, effectively balancing cold stress tolerance and photosynthetic productivity, whereas <12 °C temperatures induce energy-intensive stress responses at the expense of growth. This temperature-dependent trade-off underscores the importance of thermal regulation in maximizing both yield and stress tolerance in early-season Chinese cabbage production.

### 3.9. Quantitative Real-Time PCR (qRT-PCR) Validation

To assess the reliability of the RNA-seq data, 15 differentially expressed genes (DEGs) were randomly selected for validation via quantitative real-time PCR (qRT-PCR). Gene-specific primers were designed using Primer Premier 5.0 software (Premier Biosoft International, Palo Alto, CA, USA) (Table S3). The qRT-PCR results closely mirrored the RNA-seq expression profiles, with consistent expression trends observed across all tested genes (Figure S3). A high correlation coefficient (R^2^ = 0.8853) between the two datasets further confirmed the robustness and accuracy of the transcriptome data.

## 4. Discussion

Cold temperatures significantly impact the growth, development, and quality of Chinese cabbage, a widely cultivated leafy vegetable in the world. Exposure to low temperatures, especially frost or prolonged cool conditions, can induce physiological stress in the plant, affecting its cell metabolism, water retention, and photosynthetic efficiency. Understanding how Chinese cabbage responds to cold stress is crucial for optimizing its cultivation, particularly in regions with fluctuating temperatures or during the early or late growing seasons. Moreover, cold tolerance is often linked to genetic variation, which can be leveraged in breeding programs to develop more cold-stress-resilient varieties. Here, we have employed RNA-seq-based method to conduct a comparative transcriptome analysis of Chinese cabbage (LY2 variety) leaf samples from plants grown under 6, 9, 12, and 15 °C to identify key genes involved in Chinese cabbage cold stress response. Further, we integrated RNA-seq analysis with WGCNA to identify key regulatory (hub) genes and gene co-expression networks associated with Chinese cabbage cold stress response.

### 4.1. Chinese Cabbage Differential Responses to Low Temperature (Cold Stress) Gradient at the Phenotypic and Physiological Levels

The temperature-dependent regulation of growth and physiological processes in Chinese cabbage demonstrates a critical interplay between metabolic activities and environmental adaptation. The superior vegetative growth (leaf number, plant height, stem diameter) observed at 15 °C aligns with enhanced enzymatic activation in carbohydrate metabolism and cell expansion under moderate thermal conditions, as reported in *B. rapa* by Zhang et al. [35]. The increase in leaf area at 12–15 °C further supports the role of warmer temperatures in optimizing leaf photosynthetic surface development, which facilitates light interception and carbon assimilation. Conversely, growth inhibition at ≤9 °C reflects cold-induced suppression of meristematic activity and nutrient translocation, consistent with chilling stress responses documented in leafy vegetables by Li et al. [36].

Physiologically, the pronounced elevation of antioxidant enzymes (POD and CAT) and proline accumulation at 6 °C underscores the activation of energy-intensive stress defense mechanisms under suboptimal temperature conditions. These responses align with the “cold shock” paradigm, whereupon reactive oxygen species (ROS) overproduction necessitates up-regulated antioxidant systems to prevent membrane peroxidation [37]. The progressive decline in these stress markers at 12–15 °C indicates a metabolic shift toward growth prioritization, whereby reduced oxidative damage allows for reallocation of resources to chlorophyll synthesis and soluble sugar accumulation, a trade-off termed “photosynthetic priming” by Suzuki et al. [38]. The peak chlorophyll content and carbohydrate reserves at 15 °C likely reflect Rubisco activation and increased Calvin cycle efficiency at that temperature; these thermally regulated processes are critical for biomass accumulation [35]. Taken together, these findings reveal 15 °C as the optimal temperature for balancing cold stress tolerance and growth in spring Chinese cabbage. While 12 °C sustains moderate growth, the full growth-associated processes occur only at 15 °C, as evidenced by the simultaneous minimization of stress markers (proline, electrolyte leakage) and maximization of photosynthetic output. This aligns well with the resource allocation theory, whereupon plants under benign environment redirect energy from stress mitigation towards growth [38]. For cultivation purposes, maintaining temperatures above 12 °C during early growth stages is critical for avoiding cold stress-associated yield penalties in Chinese cabbage. Future research should explore genotype-specific thermal thresholds and the role of diurnal temperature fluctuations in modulating these physiological trade-offs.

### 4.2. Key Cold-Stress-Responsive (Hub) Genes Identified by Transcriptome Analysis and WGCNA 

Our RNA-seq transcriptome and WGCNA analyses identified several key cold-stress-responsive (hub) genes in the key modules, including photosynthesis-related, protein kinases, TFs, and “response to stress” related DEGs. Photosynthesis-related genes including the RuBisCO activase genes, photosystem I (PS I) and photosystem II (PS II) subunits proteins, and light-harvesting complex (LHC) proteins were identified across four low-temperature treatment conditions (Table S2). The plant’s ability to cope with cold stress conditions hinges on its modulation of photosynthesis involved genes, which regulate various processes such as light capture, electron transport, carbon fixation, and photosynthetic protein stabilization [9]. RuBisCO (key enzyme that catalyzes CO_2_ fixation) activation is often reduced in response to chilling stress, ultimately decreasing carbon fixation efficiency. However, in Chinese cabbage, cold-responsive TFs, particularly CBFs, can modulate the expression of RuBisCO activase genes [39], which may help the enzyme recover its activity under cold stress [40]. The regulation of RuBisCO and associated enzymes may help maintain a limited level of carbon fixation and photosynthetic output, even under adverse low-temperature conditions.

The cold stress-induced reduction in membrane fluidity may impair PSI and PSII functioning, resulting in diminished light capture and electron transport [41]. PSII is particularly vulnerable to cold stress, as it requires a fluid, dynamic membrane structure for efficient electron transport and photochemistry [42]. Additionally, cold-triggered alterations in membrane lipid composition and fluidity can disrupt the functioning of LHCs associated with PSII [43]. In this study LHCs and PSII-related proteins were differentially expressed in Chinese cabbage under low-temperature conditions (Table S2). While the differential expression of LHC and PSII-related genes may contribute to the observed decrease in light capture efficiency [44], the modulated expression of specific chlorophyll-binding proteins in cold-acclimated plants can optimize light absorption and minimize energy loss in low-temperature environments [39].

Protein kinases mediate cold signal transduction in plants [45,46]. Here, several protein kinase genes, including *BraA06g038980.3.1C*, *BraA01g042790.3.1C*, *BraA08g024580.3.1C,* and *BraA01g024410.3.1C,* were identified in key modules as hub genes responding to cold stress (Table S2). The cold stress signal may be sensed by membrane proteins, possibly plasma membrane (PM)-localized receptor-like kinases (RLKs) and histidine kinases (HKs). The PM-localized cytoplasmic receptor-like COLD-RESPONSIVE PROTEIN KINASE1 (CRPK1) phosphorylates the cytosolic 14-3-3 proteins and induces their nuclear import to fine-tune C-REPEAT/DRE BINDING FACTORs (CBFs) attenuation and stability, thereby facilitating PM-to-nucleus cold stress signal transduction [47]. However, it is not clear to which kinase families the protein kinases identified in this study belong to; therefore, further functional verification of these individual genes will clarify their exact families and roles in low-temperature stress response.

Meanwhile, TFs crucially orchestrate plant responses to abiotic stress, including cold stress. Cold exposure activates a complex network of TFs to regulate the expression of specific stress-responsive genes. In this study, a large number of TFs, including CBFs, MYBs, WRKY, AP2, HD-ZIP, bHLH, etc., were identified to be differentially expressed in response to cold stress in Chinese cabbage (Table S2). These TFs mediate several physiological processes such as osmoprotectant accumulation, antioxidant defense and metabolic adjustments. CBFs are a central TF family involved in cold stress response [48]; the CBF pathway is highly conserved across several plant species, with its activation being vital for inducting the expression of cold-stress-responsive genes [49]. MYB TFs modulate the expression of cell wall biosynthesis- and secondary metabolite (phenolics, flavonoids, etc.) biosynthesis-related genes, as well as stress-related proteins production. Low-temperature stress actuates specific MYB-related genes that enhance the accumulation of flavonoids and phenolic compounds, which act as antioxidants, protecting the plant against oxidative cell damage [50]. HD-ZIP genes participate in cold stress response by regulating cell membrane stability and ROS scavenging [51]. Wheat HD-ZIP gene *TaHDZipI-2* overexpressed in barley influences cold stress tolerance by controlling the cold-regulated expression of gene (COR gene) *HvTMC-AP3* [52]. *AtHB13* is also involved in low-temperature response by promoting the accumulation of antifreeze proteins and enhancing cell membrane stability [53]. Basic/helix–loop–helix (bHLH) TF genes participate in several plant metabolic and physiological processes, including growth and morphogenesis, and secondary metabolism [54,55]. Additionally, they regulate the cold signaling pathway by triggering CBF expression [56]. In addition, bHLH TFs cross-talk with other TF family genes such as *WRKY*s, *b-ZIP*, *AP2/EREBP, etc., to* modulate the cold stress signaling pathway [56,57]. It might be likely that the specific HD-ZIP and bHLH TF genes identified in the current study play similar roles and may require further verification in the future.

Cold stress not only triggers individual TFs activation, but involves extensive cross-talks among different signaling pathways. For instance, the CBF and ABA signaling pathways interact via AREBs, yielding coordinated regulation of cold-stress-responsive genes [58]. Similarly, MYB and NAC TFs cooperate to enhance the expression of stress-related genes and fine-tune cold stress response [59]. This cross-talk helps Chinese cabbage to integrate various abiotic stress (e.g., cold, drought, anoxia, etc.) signals and adjust its metabolic and physiological processes accordingly. Taken together, these TF families, acting in concert, essentially regulate the cold stress response in Chinese cabbage, by modulating the expression of a broad array of genes related to osmotic regulation, antioxidant defense, and energy metabolism, among other processes, which helps plants adapt to low-temperature stress conditions.

### 4.3. Analysis of Module–Trait Association and the Expression Patterns of Key Cold-Responsive Genes Identified in the Special Modules

To dissect the molecular networks involved in vernalization response under varying temperature conditions, WGCNA identified several gene co-expression modules tightly associated with both transcriptional regulation and phenotypic traits. Among the 18 identified modules, turquoise (M1), tan (M6), blue (M2), and black (M3) modules emerged as key clusters potentially involved in vernalization-related processes due to their expression dynamics and strong trait correlations (Figure 7).

M1, the largest module containing 3764 DEGs and 227 transcription factors, exhibited exclusive high expression at 15 °C, a temperature often associated with vernalization effects in Brassica crops. The GO enrichment terms in this module, such as vesicle-mediated transport and endomembrane system (Table 3), suggest active intracellular signaling and protein trafficking during the vernalization phase. Furthermore, M1 showed significant positive correlations with plant height (PH; r = 0.88, *p* = 1 × 10^−4^), stem diameter (SD; r = 0.94, *p* = 7 × 10^−4^), and soluble sugar content (SSC; r = 0.91, *p* = 4 × 10^−5^), indicating that it likely contributes to vegetative growth and carbohydrate accumulation during or after vernalization.

The tan module (M6) was notable for its negative correlation with growth-related traits (LN, PH, SD, LA), yet showed positive associations with physiological markers of cold stress, including POD (r = 0.85, *p* = 4 × 10^−4^), CAT (r = 0.93, *p* = 1 × 10^−5^), proline content (r = 0.90, *p* = 6 × 10^−5^), and REC (r = 0.87, *p* = 3 × 10^−4^) (Figure 7). This indicates that tan module genes are likely activated as a part of a stress adaptation mechanism rather than developmental progression. Importantly, tan module genes were enriched in ribosome biogenesis and rRNA metabolic processes (Table 3), essential for protein synthesis under changing temperature conditions.

M2, rich in 209 TFs, displayed higher expressions at 9 °C and 15 °C, and lower expressions at 6 and 12 °C. Functional annotation showed enrichment in small molecule metabolic processes, response to abiotic stimuli, and ion transport (Table 3), suggesting this module might regulate internal metabolic reprogramming needed for vernalization. Moreover, M2 showed a strong positive correlation with REC (r = 0.92, *p* = 3 × 10^−5^) (Figure 7), supporting its role in abiotic stress signaling during cold exposure.

Conversely, the black module (M3) was negatively correlated with proline (r = −0.85, *p* = 4 × 10^−4^) and REC (r = −0.89, *p* = 1 × 10^−4^) (Figure 7), which might indicate that these genes are down-regulated as a protective mechanism to modulate stress sensitivity. This module, expressed predominantly at 6 °C, was functionally enriched in transport and endoplasmic reticulum functions (Table 3), which may reflect early cellular stress response rather than direct vernalization regulation.

In summary, the identification of key modules through WGCNA offers a deeper understanding of the molecular mechanisms governing cold tolerance and vernalization in Chinese cabbage. Modules such as M1, M2, M3, and M6, along with the associated hub genes, provide a valuable resource for investigating the genetic pathways that regulate cold stress responses and vernalization. Further studies focusing on validating the function of these hub genes and understanding their interaction in the context of vernalization and cold stress will be crucial for advancing crop improvement strategies.

### 4.4. DEGs Related to ‘Response to Abiotic Stress’ Identified Under Cold Conditions

Here, we identified several DEGs that were annotated to “response to abiotic stress” (Table 3), including abscisic acid (ABA)-related, dehydrins (DHNs), late embryogenesis abundant (LEA), heat shock proteins (HSPs), peroxidases, and TFs among others (Table S3). ABA crucially mediates plant cold stress response by regulating the expression of cold-responsive (COR) genes and modulating several other protective mechanisms [60]. In addition to gene regulation, ABA helps in maintaining water balance during cold stress by promoting stomatal closure, which reduces water loss through transpiration [61]. This is particularly important during freezing conditions, as excessive water loss can lead to dehydration and cellular damage. Cold temperatures often lead to ROS generation, which can cause oxidative damage to lipids, proteins, and nucleic acids [62]. To counteract this, Chinese cabbage activates a range of antioxidant metabolic pathways. Peroxidases, localized in the vacuole and the cell wall, act as the first line of cell defense by detoxifying ROS-generated hydrogen peroxide [63].

Cold-induced expression of specific genes leads to the production of cold-responsive proteins such as DHNs and LEA proteins, which are involved in protecting cells from dehydration and membrane damage [64,65]. These proteins are often regulated by TFs such as C-repeat binding factors (CBFs) and ABF/AREB. The CBF pathway, in particular, is activated by low temperatures and regulates the expression of cold-responsive genes, including those encoding for enzymes involved in sugar biosynthesis and stress-related proteins [66,67]. Additionally, HSPs respond to cold stress by helping to refold of denatured proteins and preventing protein aggregation [68]. The enhanced expression of these cold-responsive proteins ensures cellular integrity and survival under cold conditions [69]. Taken together, our results showed that under cold stress conditions, the Chinese cabbage enhanced its cellular redox homeostasis and activated cold response genes to regulate the steady state of ROS and resist cold stress.

### 4.5. Significantly Enriched Metabolic Pathways of DEGs Under Cold Stress

Multiple metabolic processes are affected by cold stress. Here, our KEGG pathway enrichment analysis revealed key metabolic pathways that responded to low-temperature stress, including those related to “metabolic pathways” and “secondary metabolites biosynthesis” that became highly enriched in A6_vs_A15 and A12_vs_A15 comparison groups, “starch and sucrose metabolism” that were enriched in the A12_vs_A15 group, and “ribosome” and “ribosome biogenesis” that were significantly enriched in the A9_vs_A15 comparison group (Figure 4). Secondary metabolites are non-essential compounds for basic plant growth but provide adaptive roles under stress conditions, and the accumulation of secondary metabolites such as phenolic compounds, flavonoids, and glucosinolates is notably influenced by cold exposure [70,71]. These compounds contribute to both antioxidant defense and plant growth regulation, via stress-responsive gene expression modulation [72]. Cold stress often triggers phenolic compound biosynthesis in plants, which acts as antioxidants to protect cells against ROS-induced oxidative damage. Flavonoids also crucially stabilize cell membranes, protecting cells against cold-induced dehydration [73,74]. Thus, the secondary metabolites biosynthesis pathway was significantly enriched with key cold-stress-responsive DEGs.

Starch and sucrose metabolism are central in cellular energy provision, which contributes to plants tolerance to stressful conditions [75]. Under cold exposure, Chinese cabbage undergoes adaptive shifts in starch metabolism to balance energy storage and utilization. Under moderate cold stress, starch biosynthesis is enhanced, possibly as a response to reduced photosynthetic activity [76]. Starch is broken down into simpler sugars such as glucose, through the action of enzymes such as α-amylase and β-amylase; the resulting glucose can be directly used for energy production or further processed into other metabolites [77]. Starch mobilization is regulated by the balance between starch synthase and starch phosphorylase activities, which are sensitive to temperature changes [76]. Cold temperatures often lead to reduced metabolic activity, yet Chinese cabbage must balance energy production to support stress responses; thus, the starch and metabolism pathway was significantly enriched in the current study.

Ribosome biosynthesis is a highly energy-intensive process, and cold stress causes a reduction in ribosome biogenesis to conserve cellular energy resources. During cold stress, transcription of ribosomal RNA (rRNA) genes is often down-regulated, leading to reduced ribosome biosynthesis. Key regulatory pathways, such as the TOR (Target of Rapamycin) signaling pathway, that regulates cellular cold stress response adjustments, via ribosome biogenesis modulation, become actuated [78]. Taken collectively, secondary metabolites biosynthesis, starch and sucrose metabolism-mediated energy production, and reduced ribosome biogenesis are critical for helping plants survive cold stress conditions.

### 4.6. Nonlinear Transcriptomic Responses to Moderate Cold Stress Reveal Complex Regulatory Mechanisms

The comparison between the 9 °C and 15 °C treatments (A9 vs. A15) provided key evidence indicating a nonlinear relationship between temperature and gene expression patterns during vernalization in Chinese cabbage. Although extreme temperature conditions (6 °C and 15 °C) yielded the greatest difference in DEG numbers, intermediate temperatures (such as 9 °C) exhibited unique transcriptional responses that differed from a simple linear model. Specifically, the number of down-regulated genes at 9 °C exceeded up-regulated ones, suggesting a possible suppression or modulation of certain metabolic and growth-related pathways under intermediate temperature conditions. Moreover, KEGG pathway analysis identified distinct enrichments in pathways related to ribosome biogenesis and ribosomal activity specifically at 9 °C, implying enhanced translational regulatory processes possibly aimed at balancing growth and stress responses [27]. The complexity of this intermediate-temperature response is further supported by K-means clustering, which highlighted specific gene clusters (such as cluster 17) which maintained high expression at temperatures of 9 °C and above, and were significantly enriched in metabolic processes related to lipid biosynthesis and organic acid metabolism. Similarly, WGCNA modules like M2, significantly enriched in small molecule metabolic processes and ion transport, further underscore the intricate regulatory mechanisms in operation at moderately cold temperatures. These observations collectively suggest that the molecular mechanisms underlying vernalization responses involve a sophisticated interplay between stress perception, metabolic reprogramming, and growth modulation, reflecting plant adaptive strategies to manage energetic and physiological trade-offs rather than following a simple linear temperature-dependent pattern.

## 5. Conclusions

In this study, transcriptomic analysis was conducted on Chinese cabbage under four growth conditions of 6, 9, 12, and 15 °C. A total of 1832 DEGs were identified in three comparison groups, 5452, 1861, and 752 DEGs were specifically expressed in the A6_vs_A15, A9_vs_A15, and A12_vs_A15 groups, respectively. KEGG enrichment analysis showed that pathways related to metabolic processes were significantly enriched in three groups, and the biosynthesis of secondary metabolites was highly enriched in the four comparison groups A6_vs_A9, A6_vs_A12, A6_vs_A15, and A12_vs_A15. Cluster analysis divided DEGs into different gene clusters based on their expression levels, with eight gene clusters reaching a significant level of difference. Enrichment analysis of gene cluster 2 showed that these genes were significantly enriched in response to abiotic stress. WGCNA divided DEGs into 18 modules, and GO enrichment analysis showed that M7 DEGs were significantly enriched in response to temperature stimuli, making M7 a key candidate gene cluster for Chinese cabbage’s tolerance to vernalization. Hub genes analysis identified 650 highly connected genes, including 39 transcription factors such as MYB44, bZIP9, bHLH, AP2, and Trihelix family members. Other cold-stress-responsive genes identified as core regulators include photosynthesis-related, peroxygenase, several protein kinases, TFs, and “response to stress” related DEGs. The integration of RNA-seq and WGCNA, together with phenotypic analysis, revealed a robust, temperature-dependent cold stress response in Chinese cabbage. The identified key genes and metabolic pathways can serve as valuable genetic resources or selection targets for future targeted cloning and downstream analysis research.

## Figures and Tables

**Table 1:** Summary details of the RNA-seq results for the eleven leaf samples.

Sample	Raw Reads	Clean Reads (%)	Unique_Mapped (%)	Multiple_Mapped (%)	AF_Q30 (%)	AF_GC (%)
A6-1	42,083,140	41,870,984 (99.50%)	37,672,913 (90.24%)	998,937 (2.39%)	5,791,987,638 (92.70%)	2,988,649,887 (47.84%)
A6-2	43,646,058	43,468,442 (99.59%)	39,461,219 (90.99%)	1,074,605 (2.48%)	6,073,563,050 (93.67%)	3,098,203,406 (47.78%)
A6-3	41,728,384	41,555,956 (99.59%)	37,723,296 (90.95%)	1,029,352 (2.48%)	5,840,601,159 (94.10%)	2,947,541,057 (47.49%)
A9-1	43,316,474	43,129,816 (99.57%)	38,905,260 (90.40%)	1,019,417 (2.37%)	6,028,558,747 (93.64%)	3,056,572,130 (47.48%)
A9-2	46,400,400	46,207,588 (99.58%)	41,657,360 (90.39%)	1,097,035 (2.38%)	6,479,754,563 (93.93%)	3,279,608,319 (47.54%)
A9-3	49,023,646	48,804,126 (99.55%)	44,137,405 (90.66%)	1,171,789 (2.41%)	6,781,477,998 (93.13%)	3,464,902,473 (47.58%)
A12-1	45,255,406	45,082,436 (99.62%)	40,820,878 (90.84%)	1,238,429 (2.76%)	6,270,007,583 (93.18%)	3,232,348,084 (48.04%)
A12-2	37,078,896	36,942,084 (99.63%)	33,560,542 (91.11%)	992,795 (2.70%)	5,171,610,430 (93.78%)	2,640,163,034 (47.88%)
A12-3	47,484,122	47,269,836 (99.55%)	42,801,187 (90.84%)	1,282,488 (2.72%)	6,595,279,562 (93.51%)	3,379,144,523 (47.91%)
A15-1	43,695,692	43,510,072 (99.58%)	39,363,393 (90.68%)	1,200,329 (2.77%)	6,097,045,076 (93.84%)	3,102,716,312 (47.75%)
A15-3	53,561,126	53,324,268 (99.56%)	48,169,587 (90.58%)	1,619,417 (3.05%)	7,451,366,401 (93.60%)	3,805,927,059 (47.81%)

**Table 2:** Number of transcription factors identified in different modules.

Module	TF Number	Module	TF Number
turquoise (M1)	227	sienna3 (M10)	28
blue (M2)	209	paleturquoise (M11)	27
black (M3)	189	lightsteelblue1 (M12)	24
brown (M4)	128	mediumpurple3 (M13)	19
brown4 (M5)	93	floralwhite (M14)	11
tan (M6)	68	white (M15)	8
darkgrey (M7)	47	bisque4 (M16)	8
lightcyan1 (M8)	43	ivory (M17)	6
darkgreen (M9)	33	navajowhite2 (M18)	4

**Table 3:** GO Enrichment Analysis of DEGs in Special Modules.

Module	GO ID	GO Enrichment Terms	Gene Numbers	*p*-Value	FDR
M1	GO:0016192	vesicle-mediated transport	157	1.00 × 10^−11^	1.00 × 10^−9^
	GO:0012505	endomembrane system	267	1.00 × 10^−11^	1.00 × 10^−9^
	GO:0005515	protein binding	846	1.00 × 10^−11^	1.00 × 10^−9^
	GO:0070647	protein modification by small protein conjugation or removal	117	1.00 × 10^−11^	1.00 × 10^−9^
	GO:0005794	golgi apparatus	127	1.00 × 10^−11^	2.00 × 10^−6^
M2	GO:0044281	small molecule metabolic process	504	1.00 × 10^−11^	1.00 × 10^−8^
	GO:0009628	response to abiotic stimulus	177	1.00 × 10^−11^	1.00 × 10^−6^
	GO:0006811	ion transport	233	1.00 × 10^−11^	1.60 × 10^−5^
	GO:0010035	response to inorganic substance	102	1.00 × 10^−11^	2.40 × 10^−5^
M3	GO:0051179	localization	585	1.00 × 10^−11^	2.00 × 10^−9^
	GO:0005783	endoplasmic reticulum	119	1.00 × 10^−11^	2.10 × 10^−5^
	GO:0006810	transport	544	1.00 × 10^−11^	2.50 × 10^−5^
M4	GO:0006412	translation	454	1.00 × 10^−11^	1.00 × 10^−9^
	GO:0043043	peptide biosynthetic process	460	1.00 × 10^−11^	1.00 × 10^−9^
	GO:0006518	peptide metabolic process	469	1.00 × 10^−11^	1.00 × 10^−9^
M6	GO:0042254	ribosome biogenesis	107	1.00 × 10^−11^	1.00 × 10^−9^
	GO:0022613	ribonucleoprotein complex biogenesis	109	1.00 × 10^−11^	1.00 × 10^−9^
	GO:0016072	rRNA metabolic process	70	1.00 × 10^−11^	1.00 × 10^−9^
M7	GO:0009266	response to temperature stimulus	21	4.00 × 10^−6^	8.47 × 10^−3^
	GO:0009628	response to abiotic stimulus	39	1.10 × 10^−5^	1.18 × 10^−2^
	GO:0019685	photosynthesis, dark reaction	4	3.20 × 10^−5^	1.76 × 10^−2^
	GO:0019253	reductive pentose-phosphate cycle	4	3.20 × 10^−5^	1.76 × 10^−2^
	GO:0009987	cellular process	303	1.12 × 10^−4^	4.97 × 10^−2^
M8	GO:0006412	translation	74	1.00 × 10^−11^	2.00 × 10^−5^
	GO:0006518	peptide metabolic process	79	1.00 × 10^−11^	9.70 × 10^−5^
	GO:0006518	peptide metabolic process	79	1.00 × 10^−11^	9.70 × 10^−5^
M10	GO:0010812	negative regulation of cell–substrate adhesion	3	2.80 × 10^−5^	1.86 × 10^−2^
	GO:0010810	regulation of cell–substrate adhesion	3	4.50 × 10^−5^	1.86 × 10^−2^
	GO:0051270	regulation of cellular component movement	5	5.20 × 10^−5^	1.86 × 10^−2^

## Data Availability

Data is contained within the article.

## Supplementary Materials

The following supporting information can be downloaded at https://www.mdpi.com/article/10.3390/genes16070845/s1, Figure S1: GO enrichment analysis of specific gene clusters from K-means clustering of differentially expressed genes; Figure S2: KEGG enrichment analysis of specific gene clusters from K-means clustering of differentially expressed genes; Figure S3: Validation of RNA-seq expression data by qRT-PCR analysis. (A)Validation was performed using 15 selected DEGs. (B)The plots demonstrate the expression ratio in log scale with base of two. The X-axis indicates RNA-seq log scale; the Y-axis indicates qRT-PCR log scale. Table S1: Transcription factors detected in distinct modules using WGCNA Analysis; Table S2: Hub Genes Associated with Vernalization identified by WGCNA. Table S3: Gene specific primers used for qRT-PCR analysis.
