# Supplementary material for: Global Transcriptome and Weighted Gene Co-Expression Network Analyses of Cold Stress Responses in Chinese Cabbage
Source: Genes (Basel). 2025 Jul 20;16(7):845. doi: 10.3390/genes16070845 (PMC12294169; doi:10.3390/genes16070845)
Supplement: Supplementary file 1 [file genes-16-00845-s001.zip › Supplementary Figures.pdf]

Supplementary Figures (Total 3)

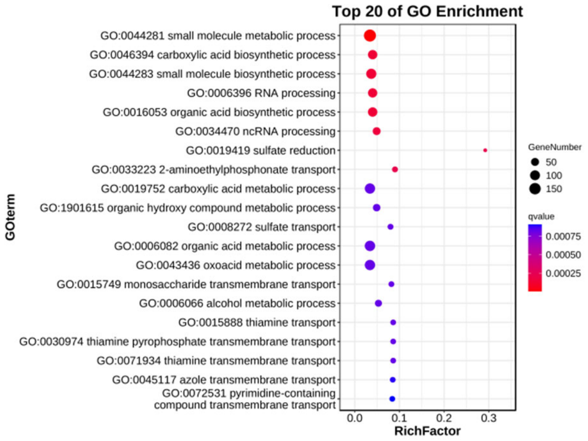

Cluster 0

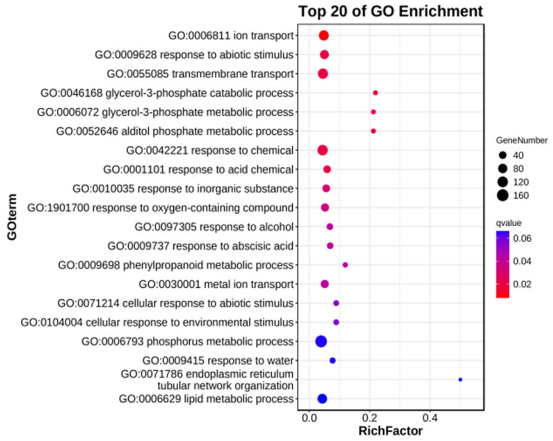

Cluster 2

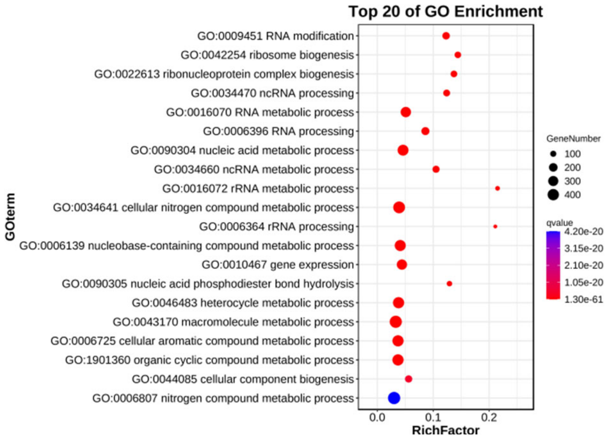

Cluster 7

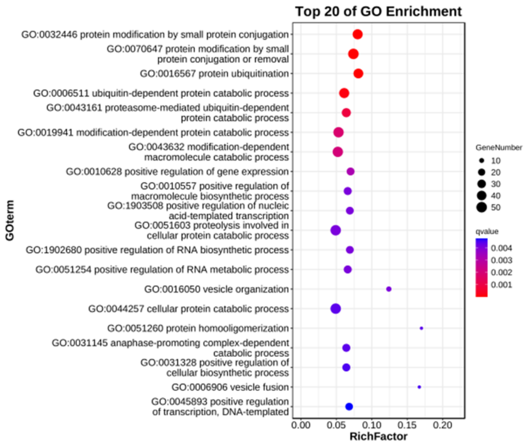

Cluster 10

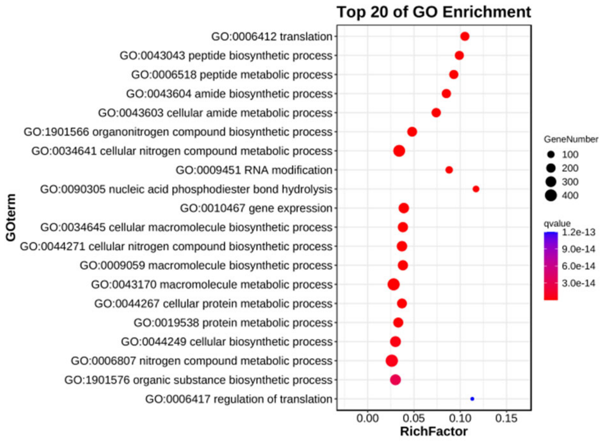

Cluster 13

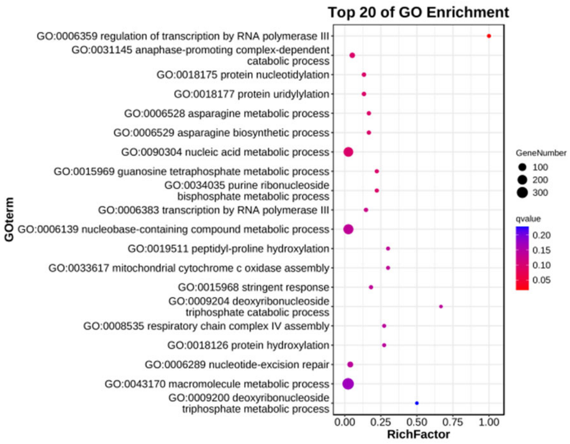

Cluster 15

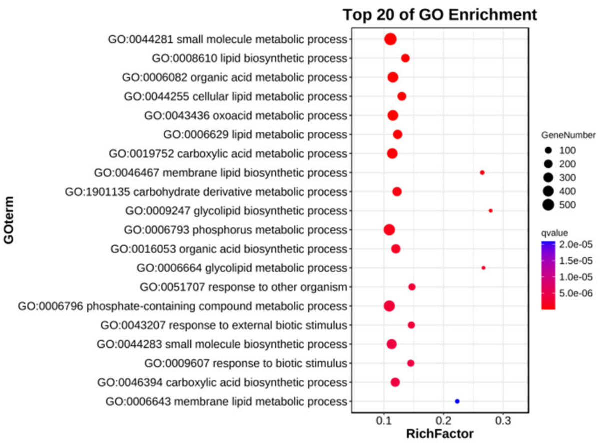

Cluster 17

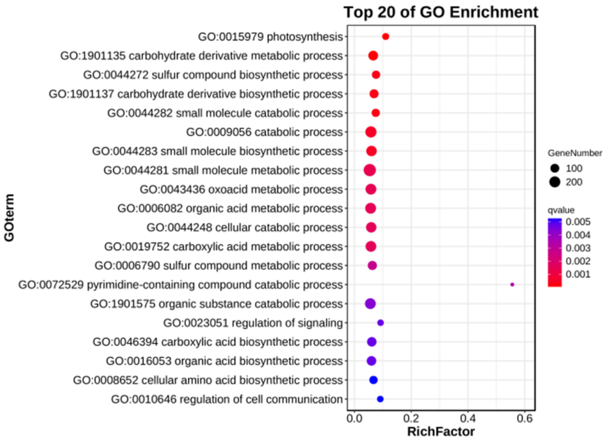

Cluster 19

Supplementary Figure S1. GO enrichment analysis of specific gene clusters from K-means clustering of differentially expressed genes

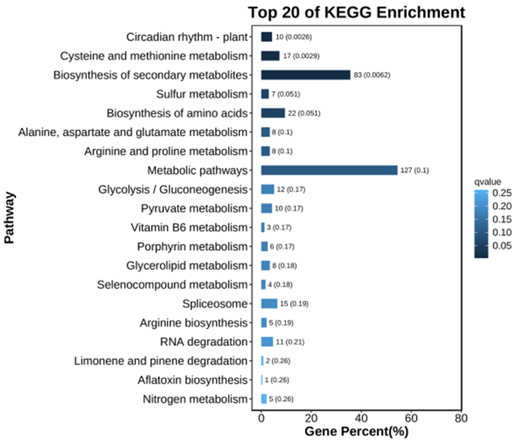

Cluster 0

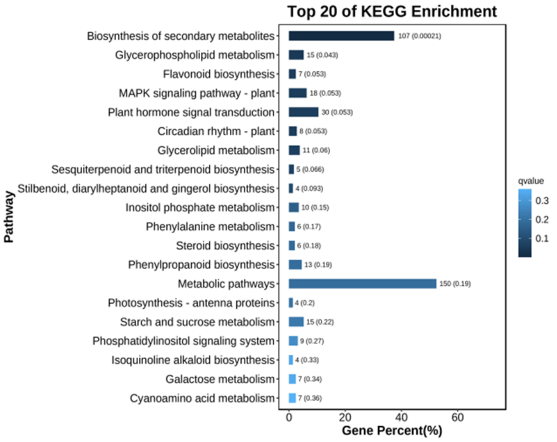

Cluster 2

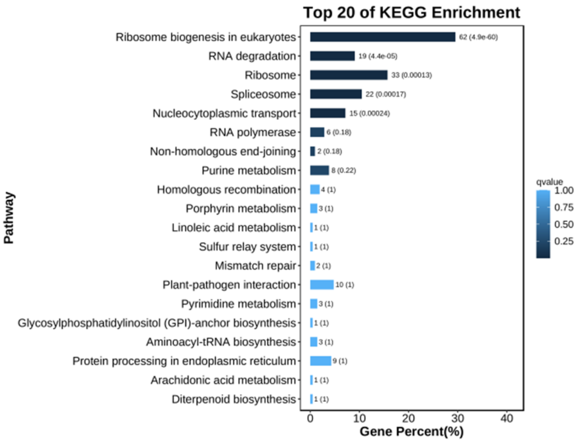

Cluster 7

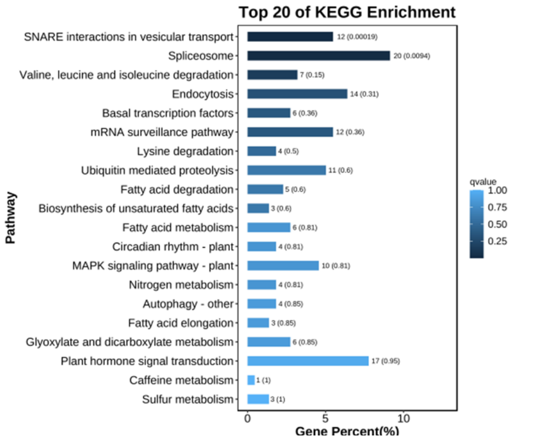

Cluster 10

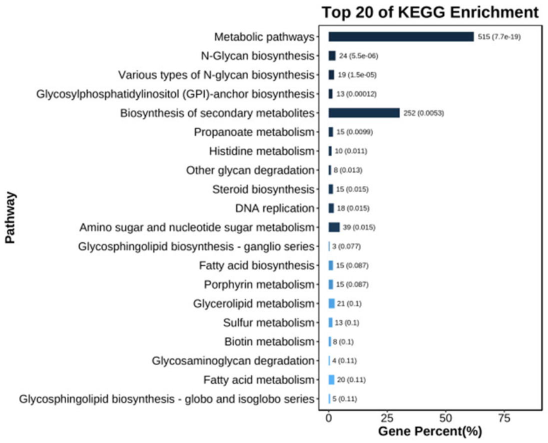

Cluster 17

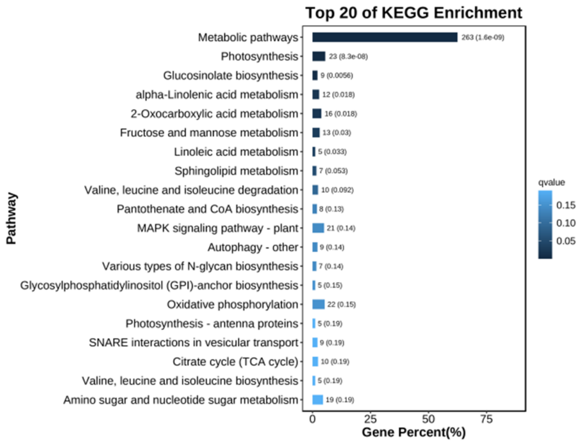

Cluster 19

Supplementary Figure S2. KEGG enrichment analysis of specific gene clusters from K-means clustering of differentially expressed genes

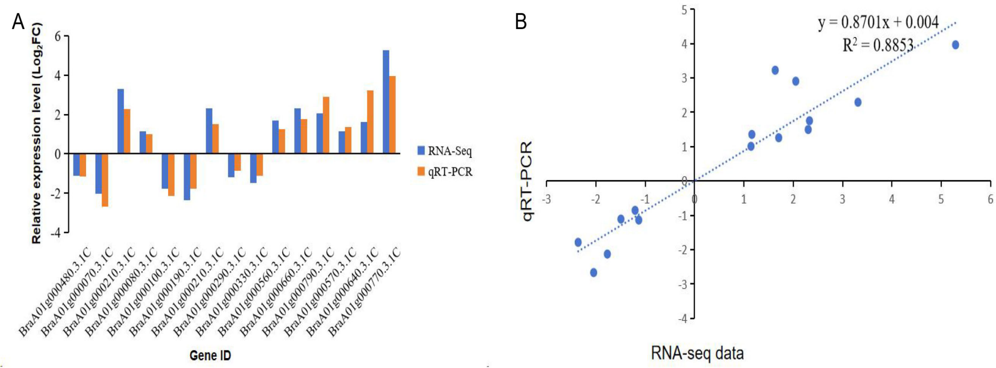

Supplementary Figure S3. Validation of RNA-seq expression data by qRT-PCR analysis. (A)Validation was performed using 15 selected DEGs. (B)The plots demonstrate the expression ratio in log scale with base of two. The X-axis indicates RNA-seq log scale; the Y-axis indicates qRT-PCR log scale.
